# Supplementary material for: Computational Identification of Novel Kir6 Channel Inhibitors
Source: Front Pharmacol. 2019 May 24;10:549. doi: 10.3389/fphar.2019.00549 (PMC6543810; doi:10.3389/fphar.2019.00549)
Supplement: Supplementary file 1 [file Presentation_1.pdf]

Supplementary Material

Supplementary Methods

Potential of mean force (PMF) and umbrella sampling

PMF $\mathcal{W}(\xi)$ describes the free energy profiles of the system along reaction coordinates ξ, which can be defined as a distance, an angle, RMSD or more complicated functions. In our case, PMF describes the free energy changes of a ligand pathway, moving from the binding site to the solvent. The mathematical expression of PMF can be found in Roux, 1995 (Roux, 1995): The PMF $\mathcal{W}(\xi)$ can be defined from the average distribution function $\langle \rho(\xi) \rangle$ in equation (1):

$$\mathcal{W}(\xi) = \mathcal{W}(\xi^*) - k_{\mathrm{B}} T ln \left[\frac{\langle \rho(\xi) \rangle}{\langle \rho(\xi^*) \rangle} \right] \quad (1)$$

where $\mathcal{W}(\xi^*)$ and $\langle \rho(\xi^*) \rangle$ are arbitrary reference values, k_{B} is the Boltzmann constant and T is the temperature. The average distribution function $\langle \rho(\xi) \rangle$ along the coordinate ξ is obtained from a Boltzmann weighted average. In case an energy barrier present, the average distribution function cannot be computed due to the lack of sampling, non-Boltzmann sampling so called umbrella sampling (US) method can be applied to obtain PMF.

With US technique, the reaction coordinates space is divided into a series of windows. For each window, simulations are run with applying a harmonic biasing potential to the system, to ensure that high-energy regions are sufficiently sampled. The windows are well sampled when the distribution function at two adjective windows have good overlap (see histograms in Supplementary Figure S4, below). An unbiased PMF $\mathcal{W}_i(\xi)$ for each window i is recovered from the biased simulations following equation (2):

$$\mathcal{W}_i(\xi) = \mathcal{W}(\xi^*) - k_{\mathrm{B}} T ln \left[\frac{\langle \rho(\xi) \rangle_i^{biased}}{\langle \rho(\xi^*) \rangle} \right] - w_i(\xi) + F_i \quad (2)$$

where $\mathcal{W}(\xi^*)$ and $\langle \rho(\xi^*) \rangle$ are arbitrary reference values, k_{B} is the Boltzmann constant and T is the temperature, $\langle \rho(\xi) \rangle_i^{biased}$ is the average of biased distribution function of each window i , $w_i(\xi)$ is the biasing harmonic potential applied to restrain the system, F_i is undetermined constant that represents the free energy associated with $w_i(\xi)$.

The unbiased PMF $\mathcal{W}(\xi)$ of all windows and the constants F_i can be computed by the weighted histogram analysis method (WHAM) (Kumar et al., 1992).

Supplementary Figures

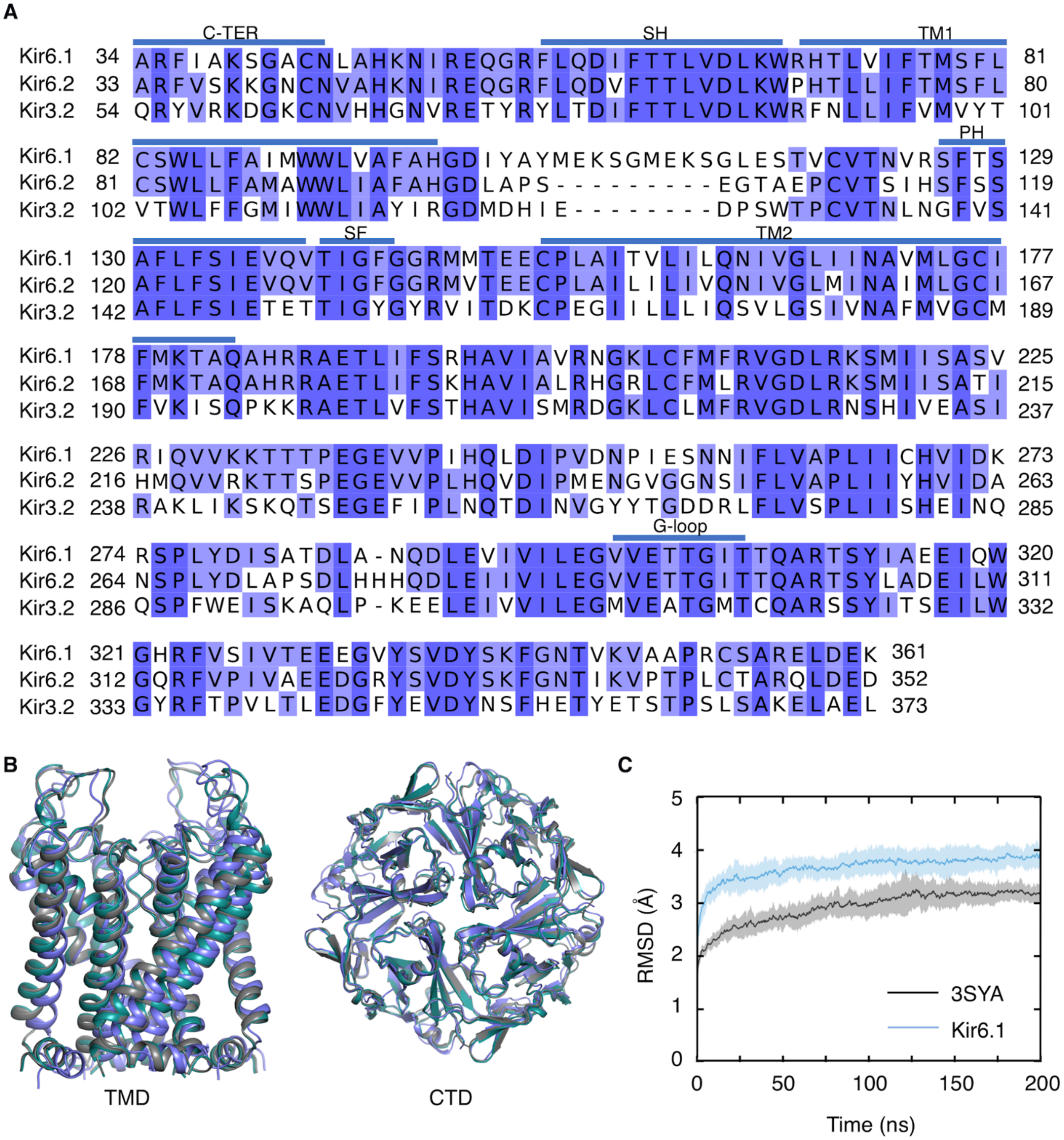

Supplementary Figure S1. Kir6.1 modelling. (A) Sequence alignment of human Kir6.1, Kir6.2 and Kir3.2 channels. **(B)** Cartoon representation of structural alignment about the TMD (side view) and the CTD (top view) of the Kir6.1 model in green, with the Kir3.2 template in grey (PDB code: 3SYA) and Kir6.2 in purple (PDB code: 6BAA). The RMSDs of structure alignments for the TMD and CTD part respectively are: 0.56 Å and 0.58 Å between Kir6.1 and Kir3.2, 0.86 Å and 1.34 Å between Kir6.1 and Kir6.2, and 0.92 Å and 1.02 Å between Kir6.2 and Kir3.2. **(C)** The average

backbone RMSDs of the Kir6.1 model and the Kir3.2 template from three independent 200 ns MD simulations, show proteins are stable.

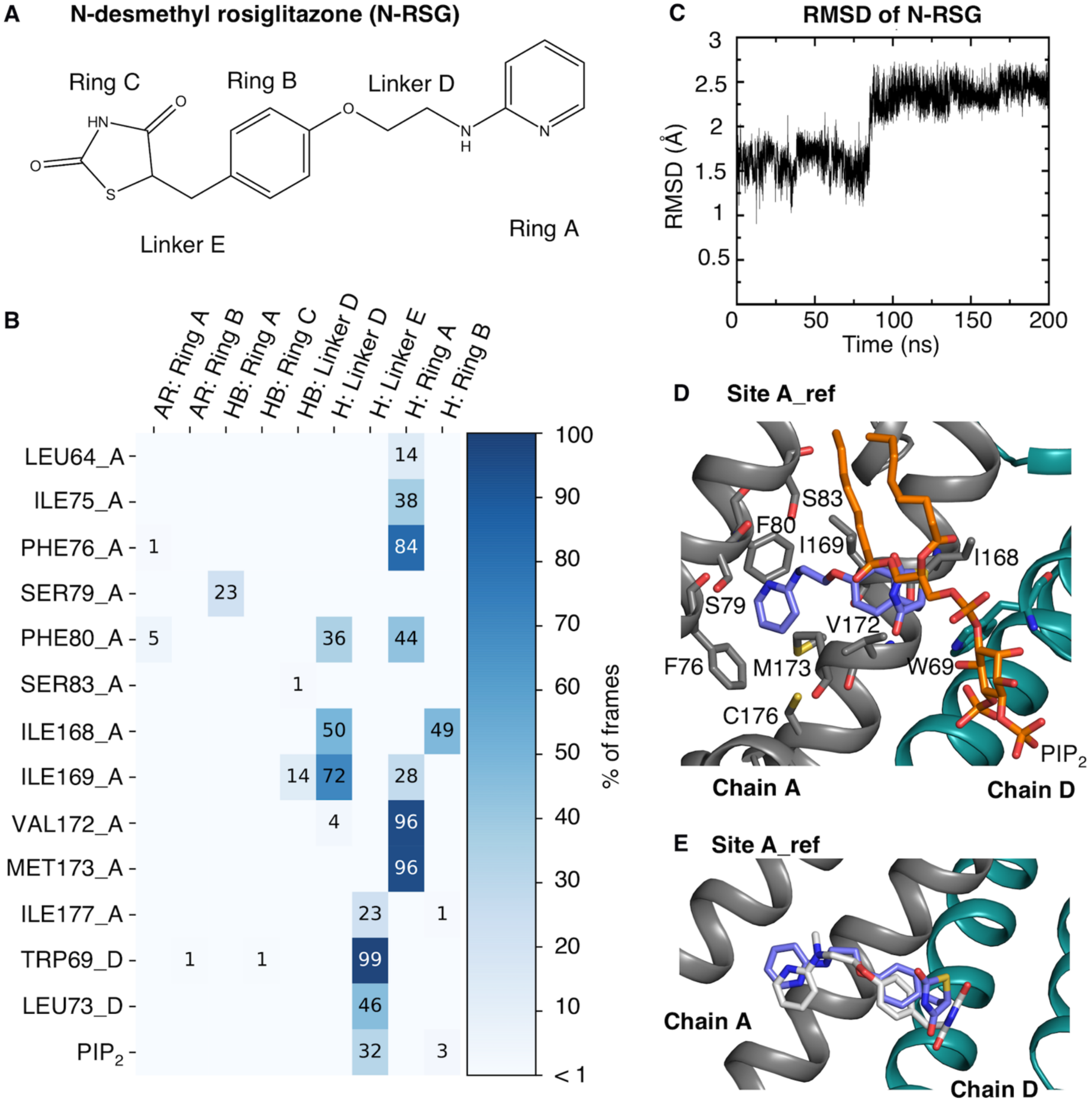

Supplementary Figure S2. N-RSG interaction with the protein and PIP_2 at binding site A_ref. **(A)** Molecular structure of N-RSG including the denotation corresponding to the interaction map. **(B)** Interaction map of N-RSG with protein and PIP_2 during 200 ns MD simulation. The matrix is coloured and numbered by the percentage of frames, in which the interactions were observed: aromatic (AR), hydrophobic (H), hydrogen bond (HB). The residues in the Kir6.1 are named by the corresponding amino acid with its residue number and chain ID (A or D). **(C)** RMSD plot of N-RSG at binding site A_ref during a 200 ns MD simulation. **(D)** Best PMF energy pose: Kir6.1 is

represented as cartoon with the two neighbouring subunits coloured in grey and green, respectively. N-RSG (purple), the surrounding residues within 3.5 Å, and the PIP_2 (orange) are shown as stick. **(E)** Best PMF energy poses of RSG (white) and N-RSG (purple) superposed at binding site A_ref.

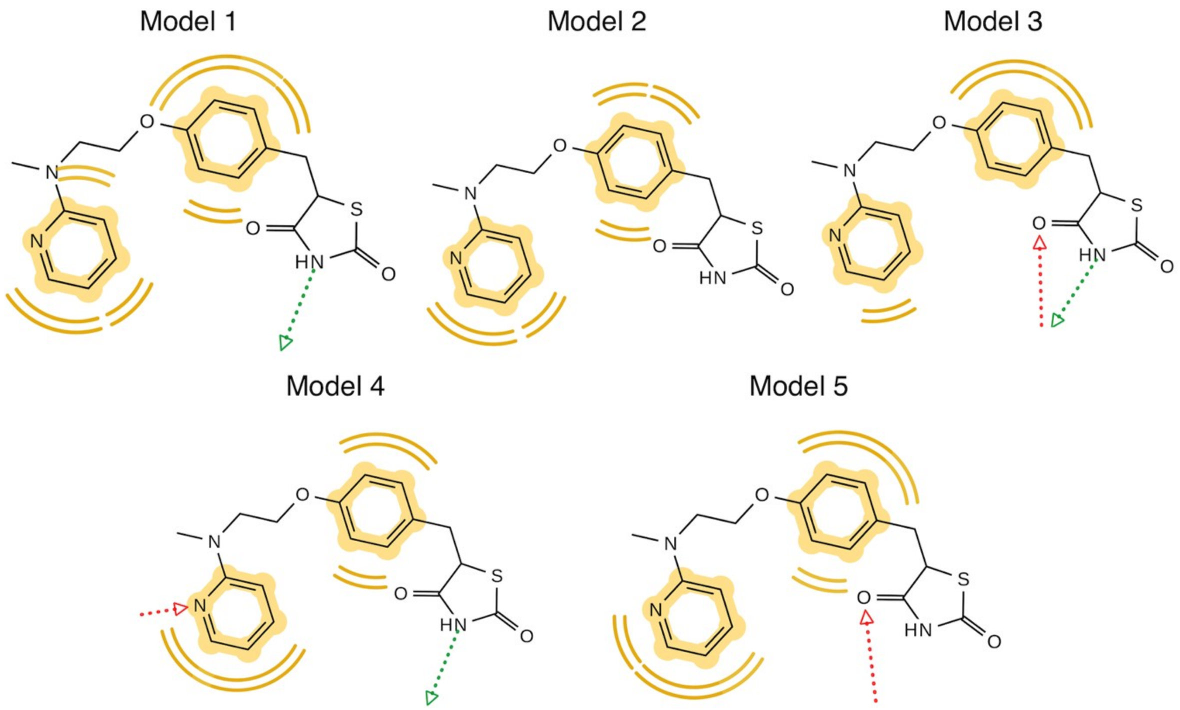

Supplementary Figure S3. Dynamic pharmacophore models of RSG derived from MD simulation at binding site A_ref. The yellow regions represent hydrophobic features. Red arrows represent hydrogen bond acceptors (HBA) and green arrows represent hydrogen bond donors (HBD).

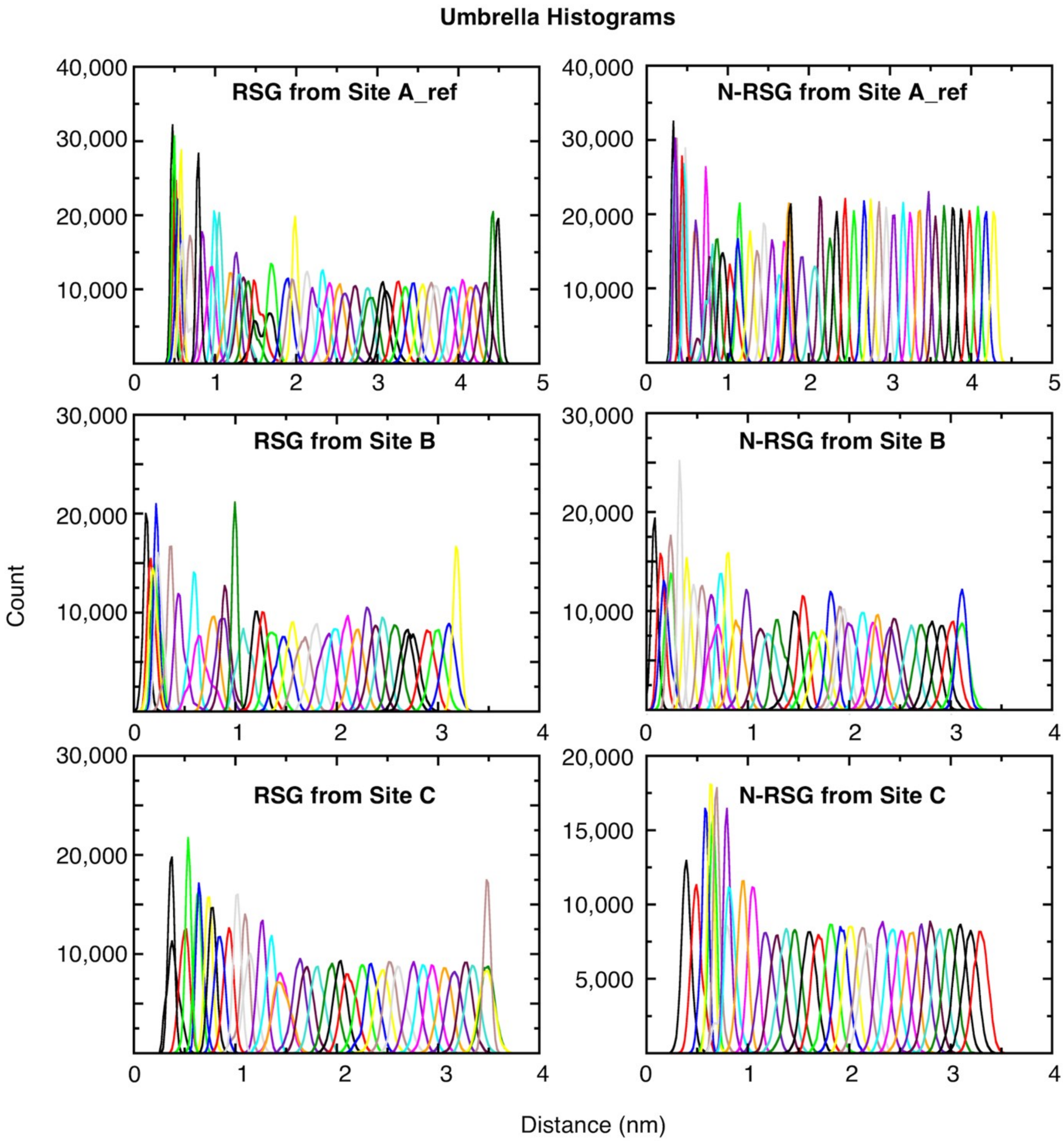

Supplementary Figure S4. US histograms of RSG and N-RSG from the three distinctive binding sites. Each plot represents the distribution function of a window, which shows good overlap. In total, 242 windows were generated with 10 ns simulation performed per window.

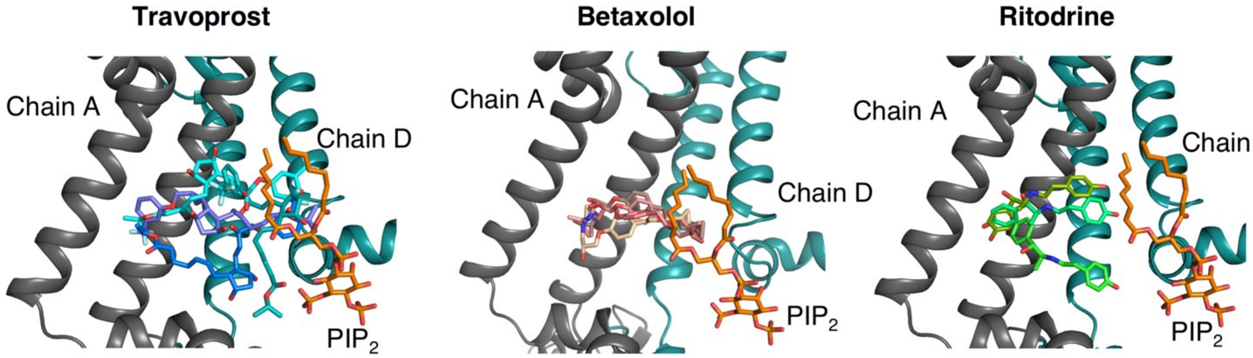

Supplementary Figure S5. Docking poses of Travoprost, Betaxolol and Ritodrine at Site A_ref. Kir6.1 is represented as cartoon with the two neighbouring subunits coloured in grey and green, respectively. The PIP_2 are shown as stick in orange. Docking poses of three ligands are represented as sticks at the binding site A_ref by different colours.

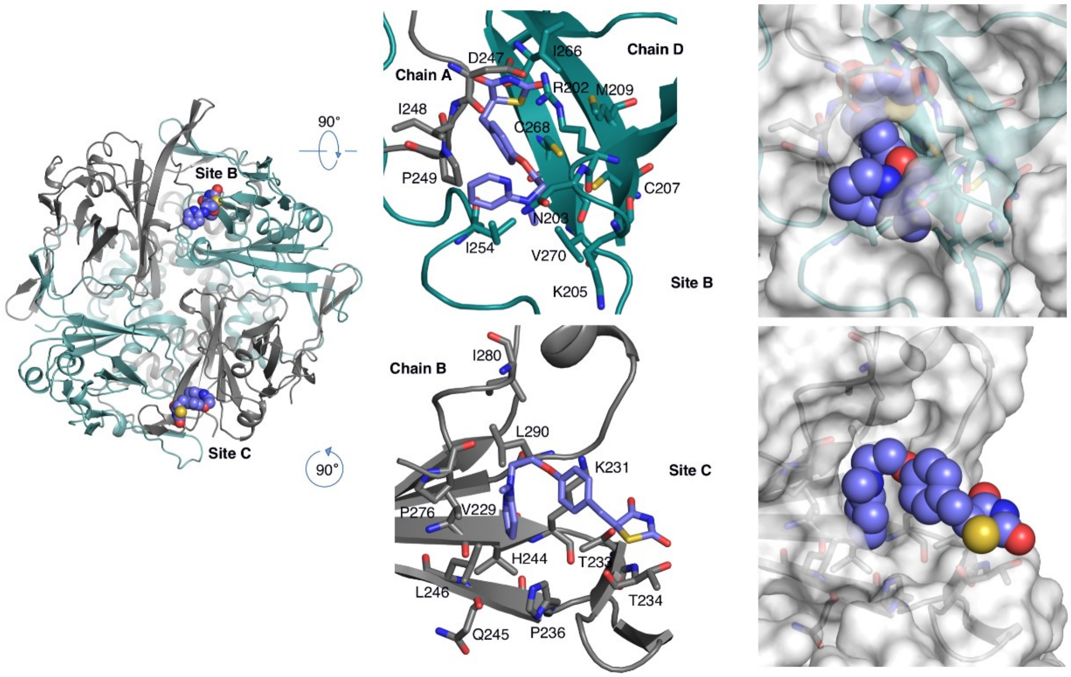

Supplementary Figure S6. Details of binding sites B and C. Left side: bottom view of the Kir6.1 channel shown as cartoon, with neighbouring subunits coloured grey and green, respectively. Middle panel: RSG is shown in stick representation, coloured in purple. Amino acids within 3.5 Å of the ligand are shown as sticks. Right panel: pockets of sites B and C are shown in semi-transparent surface representation, with the RSG ligands shown as spheres.

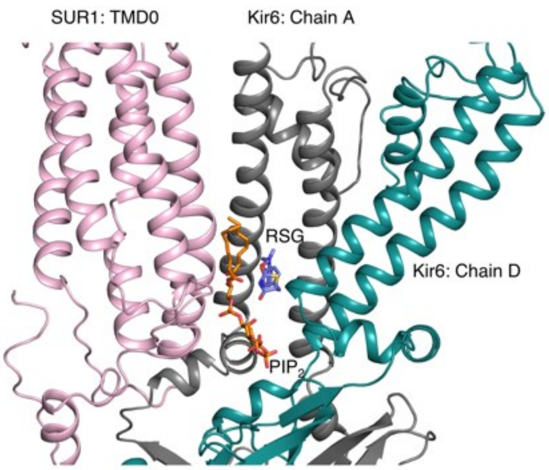

Supplementary Figure S7. Location of SUR1 relative to the proposed RSG binding site on Kir6.1. TMD0 (pink), Kir6.1 chains A (grey) and D (green) are shown as cartoon, with bound RSG (purple) and PIP2 (orange) molecules shown as sticks.

References

Kumar, S., Rosenberg, J. M., Bouzida, D., Swendsen, R. H., and Kollman, P. A. (1992). The weighted histogram analysis method for free-energy calculations on biomolecules. I. The method. *J. Comput. Chem.* 13, 1011–1021. doi:10.1002/jcc.540130812.

Roux, B. (1995). The calculation of the potential of mean force using computer simulations. *Comput. Phys. Commun.* 91, 275–282. doi:10.1016/0010-4655(95)00053-I.
